# Perceptions of an Ideal Point-of-Care Test for Sexually Transmitted Infections – A Qualitative Study of Focus Group Discussions with Medical Providers

**DOI:** 10.1371/journal.pone.0014144

**Published:** 2010-11-30

**Authors:** Yu-Hsiang Hsieh, M. Terry Hogan, Mathilda Barnes, Mary Jett-Goheen, Jill Huppert, Anne M. Rompalo, Charlotte A. Gaydos

**Affiliations:** 1 Department of Emergency Medicine, Johns Hopkins University School of Medicine, Baltimore, Maryland, United States of America; 2 Division of Infectious Diseases, Johns Hopkins University School of Medicine, Baltimore, Maryland, United States of America; 3 Division of Adolescent Medicine, Cincinnati Children's Hospital Medical Center, Cincinnati, Ohio, United States of America; 4 Division of Adolescent Medicine, the University of Cincinnati College of Medicine, Cincinnati, Ohio, United States of America; Tulane University, United States of America

## Abstract

**Background:**

A point-of-care test (POCT) for sexually transmitted infections (STIs), which offers immediate diagnosis resulting in patients receiving diagnosis and treatment in a single visit, has the ability to address some of the STI control needs. However, needs assessment from STI experts and end users about currently available STI POCTs and their future new development has not been evaluated since World Health Organization Sexually Transmitted Diseases Diagnostics Initiative was formed over 15 years ago. Therefore, our objective was to explore the perceptions of the ideal types of STI POCT for use in health care settings.

**Methodology/Principal Findings:**

A qualitative study, encompassing eight focus groups, was conducted from March 2008 through April 2009. Participants included 6 STD clinic directors, 63 clinicians, and 7 public health/laboratory/epidemiology professionals in the STI field. Discussion topics included currently available POCT, perceived barriers to using POCT in clinics, priority STI for the development of new POCT, and characteristics of the ideal POCT. All discussions were recorded and transcribed verbatim. Themes raised as barriers for current POCT included complexity, long time frames of the so-called “rapid” test, multiple time-driven steps, requiring laboratory technician, difficulty in reading result, interruption of workflow, unreliability, and invasiveness. *Chlamydia trachomatis* was identified as the priority organism for development of a new STI POCT. Themes indicated for the ideal POCT included rapid turnaround (up to 20 minutes), ease of use, non-invasive, accurate (preferred sensitivity and specificity in the range of high 90s), Clinical Laboratory Improvement Amendments (CLIA)-waived, user-friendly (for both patients and staff), compact, durable, and sturdy.

**Conclusions/Significance:**

Focus group discussions with STI experts and professionals highlighted chlamydia as the top priority pathogen for POCT development, and identified the qualities of new POCT for STIs. Participants endorsed ease of use, rapid turnaround and high accuracy as essential characteristics of an ideal POCT.

## Introduction

Sexually transmitted infections (STIs), caused by more than 30 different sexually transmissible microorganisms, comprise the leading group of reportable diseases in the United States each year with an annual estimate of more than 19 million new cases [1] and 9.3–15.5 billion dollars in costs [2], [3]. Current diagnostic testing algorithms result in a turn-around time of 2–14 days before laboratory test results are available to clinical providers and patients. This has contributed to low rates of patient return for test results [4], [5], reinfection of the presenting patient, and ongoing transmission of infection in the patient's partner(s). All of which exacerbate an ongoing public health problem [6], [7]. Due to the sexual nature of the transmission mode, STIs carry strong social and cultural stigma and, consequently, many cases remain undiagnosed or something to that effect. Stigma is thought to be one of most important factors for inadequate access to effective STI diagnosis and treatment [3], [8], [9], [10]. Stigma also disparately affect women, minorities, adolescents, marginalized and disenfranchised populations, the same vulnerable subgroup populations that STIs greatly impact and who often have little access to health care [11]. Therefore, an accurate STI diagnostic with rapid turn-around time can provide clinicians with specific test result and may allow directed STI treatment within a single visit. Such a rapid test could effectively curtail STI transmission especially in those vulnerable populations mentioned above.

A point-of-care test (POCT) for STI(s), also called a rapid, “bed-side”, “decentralized”, or “near-patient” test offering immediate diagnosis [12], could meet the World Health Organization Sexually Transmitted Diseases Diagnostics Initiative (WHO SDI). This initiative has identified benchmark criteria for an STI POCT as being: Affordable, Sensitive, Specific, User-friendly, Rapid and robust, Equipment-free and Deliverable to end-users (ASSURED) [13],[14]. Such assays have the ability to address some of the STI control needs [15], especially the advantage of patients' receiving diagnosis and treatment in a single visit in both STD clinic or non-STD clinic settings (e.g. emergency department or outreach mobile van). Currently available STI POCTs in clinics in U.S. include non-pathogen specific tests such as urine dipstick and Gram stain, to more pathogen specific tests such as wet mount test for vaginitis, rapid plasma reagin (RPR) and darkfield examination for syphilis, and rapid HIV test, which differs substantially from other tests due to significance of its result. However, needs assessment from STI experts and end users about currently available STI POCTs and future new development has not been conducted since WHO SDI was formed over 15 years ago [6].

Our goal was to explore the perceptions of the ideal types and characteristics for STI POCTs for use in clinics and other care settings through focus group discussions conducted with clinicians, opinion leaders, and public health professionals.

## Materials and Methods

### Ethics Statement

This study protocol was approved by Institutional Review Boards (IRBs) of The Johns Hopkins University School of Medicine and Cincinnati Children's Hospital Medical Center. Verbal informed consent was obtained from all participants involved in our study.

### Focus Group Discussions

A total of eight focus group discussions were conducted between March 2008 and April 2009. The first focus group, an opinion leader group comprised of 6 directors of U.S. STD “Model Clinics” identified by the Centers for Disease Control and Prevention (CDC), was conducted during the National CDC STD Prevention Conference in Chicago, Illinois. For the second focus group discussion, we invited 8 U.S., U.K., and Canadian STI experts who were attending the British Association of Sexual Health and HIV (BASHH)-American Sexually Transmitted Diseases Association (ASTDA) joint meeting in Brooklyn, New York. For the first and second focus group discussions, an email was sent to invite each participant. The remainder of focus group discussions included clinicians from one public STD clinic; one federally qualified health center; a U.S. community-based organization funded under the Health Center Consolidation Act which provides comprehensive primary care and preventive care; one public adolescent and young adult clinic; one academic hospital-affiliated adolescent clinic in Baltimore, Maryland; and one group of community pediatricians and one academic hospital-affiliated adolescent clinic in Cincinnati, Ohio. We recruited for focus group members from state medical societies, community clinical sites, medical teaching institutions, hospitals and other venues using advertising and personal contact.

### Participants

In total, 76 STI professionals, including 43 physicians, 20 nurse practitioners/physician assistants/registered nurses, 6 STD clinic directors, 2 public health directors, 1 public health laboratory director, 3 laboratory technicians, and 1 epidemiologist participated.

### Focus Group Discussion Topics

Trained structured-group discussion facilitators introduced the purpose of the project and briefly outlined the definition of a POCT. The topics explored were: (1) currently available POCTs, (2) perceived barriers to using POCTs in the participant's practice setting, (3) priority setting for the development of new POCT for STIs, (4) envisioned characteristics of an ideal POCT, and (5) acceptable levels of sensitivity, specificity and predictive values. In order to ensure that all participants in the focus groups understood the meaning of sensitivity, specificity, and predictive values, the facilitator defined what these terms meant before participants were asked the question regarding desirable sensitivity, specificity, and predictable values.

### Data Management and Data Analysis

Each focus group discussion session, which included responses to the focus group facilitator and interaction between participants, was recorded and transcribed verbatim in Microsoft Word [16], [17], [18]. Each verbatim transcription served as primary text document. Within each primary document, specific quotations were selected and codes were assigned to a word or phrase. Interview transcripts were read repeatedly and were systematically coded multiple times by the investigator (Y-H H and MB) to increase precision which was facilitated by using Atlas.ti software (version 6, Atlas.ti GmbH, Berlin, Germany). Consistency of coding was checked and no discrepant codes were identified. A qualitative content analysis [19] was performed to examine frequencies/patterns of recurring codes for potential conceptual categories in each primary document and all verbatim transcripts. Codes were then regrouped and indexed in order to identify salient themes on STI POCT by using Atlas.ti software. Recurring themes in relation to this topic were checked independently by a second reviewer (MB) and were compared and tested by re-reading transcripts and fine-tuning interpretations.

## Results

### Commonly used and reported POCTs for STIs

The most common currently used POCT for STIs reported from participants was the wet mount test, i.e. saline and potassium hydroxide (KOH) slide preparation of vaginal fluid for *Trichomonas vaginalis*, *candidiasis* and bacterial vaginosis detection. Rapid HIV test, urine dipstick, Gram stain, rapid syphilis test, i.e. RPR and darkfield, were also commonly available and used in the participants' clinics. Only two participants from a focus group conducted in Cincinnati stated that a rapid *T. vaginalis* test was available in their clinics. One participant from the United Kingdom reported a rapid syphilis test (Determine® Syphilis TP) available in his clinic.

### Perceived Barriers in use of currently available POCTs

Participants identified barriers to use of currently available POCTs for STIs in three main areas: (1) characteristics of the test, (2) issues related to operations of clinic or hospital and/or laboratory, and (3) issues related to patients. Some barriers mentioned intertwined at least two of these three areas. Some specific quotations from participants are presented in Table 1.

**Table 1:** Some quotes on point-of-care tests (POCTs) for sexually transmitted infections (STIs) from the participants of structured discussion sessions.

**Perceived Barriers in use of currently available POCTs**
***Characteristics of the test***
“multistep long tests aren't going to fly in our clinic. I mean, we've done studies with them to look at their response characteristic that would require hiring new staff.”
“not quite rapid”
“Difficulty of reading the results – faint, faint lines that you're not sure whether it's positive or negative or what to do about it.”
***Related to clinic operation***
“…the provider being interrupted while working with another patient just to give results…”
“the lab services are now being covered by the hospital labs. So, that's basically been a barrier to introduce new testing because they insist on higher levels of competency, competency training, foundation and each individual test has to be validated.”
***Related to patients***
“trying to see the next patient and get back to the first patient, when the first patient is waiting much too long for something that's supposed to be rapid.”
“Some patients don't wanna get stuck for blood, they don't wanna be swabbed – so lack of patient participation.”
**Characteristics of an ideal POCT for STIs**
“like a pregnancy test”
“something simple to use maybe one part as opposed to five different things”
“I don't care what it looks like as long as it works.”
“the quicker, the better”
“We wish it could be 5 minutes but that would be impossible.”
“…a CLIA-waived test would be helpful because in that sense someone else could potentially do it, even the clinician.”
“The test should not require a great amount of technical ability to allow clinicians to easily run the test. Patient should be able to do the test without assistance and techs, too.”
“have a yes or no, plus or minus when the test is done.”
“Something that stays flat would be nice. Because I am always worried with the oral test, I'm afraid I will knock it over.”
“Cheap enough that we afford it”
**Sensitivity, Specificity and Predictive Values**
“I would say close to a 100% as you can make it.”
“It could be on the same level of a screening test that usually we get.”

All focus groups concurred that the complexity of the test, such as the number and timed steps, was the major barrier for use of some currently available POCT for STIs. The complexity of the test was frequently described as too labor intensive with multiple-step procedures, time-driven steps, laboratory equipment usage, and difficulty in reading and interpreting the test results. Several respondents indicated that a POCT was supposed to be “rapid” but it was not “rapid” enough because of laboratory-driven processes. In many cases, the laboratory-driven processes were required by the hospital administration regulations, even for the Clinical Laboratory Improvement Amendments (CLIA)-waived POCTs, which are defined by the Food and Drug Administration (FDA) as simple laboratory examinations and procedures that are cleared by FDA for home use; employ methodologies that are so simple and accurate as to render the likelihood of erroneous results negligible; or pose no reasonable risk of harm to the patient if the test is performed incorrectly [20]. Some participants indicated that they believed the validity of some POCTs was questionable or the test was not reliable, so they hesitated to use them.

Another main consensus identified by participants from all but one (public adolescent and young adult clinic) focus group was the interruption of clinic workflow. Due to the time for completion of some POCT, medical providers in a clinic setting are often required to give test results to a patient while another patient is waiting to be seen. A couple of participants suggested that some clinicians were not emotionally ready to give a positive/reactive POCT result to patients in such a limited period of time, especially for those tests offered as part of a screening panel, such as a positive HIV test.

Finally, participants also suggested that some of the barriers came from the patients. Resistance to the use of POCTs from patients might be caused by the perceived invasiveness of sample collection procedures or wait time. Participants also perceived that some patients might not want to hear (or were not ready for) the immediate result of a POCT that was not primarily for the purpose of their visit that day, i.e. a screening POCT for HIV.

### Priority for Development of New POCT for STIs

*Chlamydia trachomatis* was ranked as the top priority for development of new POCT for STIs in 7 of 8 focus groups. The one group which did not choose chlamydia as the top priority did not name any particular microorganism or disease. Four of the groups named herpes simplex virus as a second priority, two groups identified *T. vaginalis*, and one group selected *Neisseria gonorrhoeae*. Other STIs mentioned in the discussions included: syphilis, HIV (for the detection of early seroconversion), “gardnerella” (bacterial vaginosis), yeast, and hepatitis B and C viruses. One participant stated “*we want it all*” and expressed a need for a multiplex platform to identify several agents. Another participant expressed that a new POCT should detect agents of common syndromes, e.g. non-gonococcal urethritis. One participant stated that the priority should focus on a test that could rule out several organisms.

### Characteristics of an Ideal POCT for STIs

The groups unanimously cited the over-the-counter home-use pregnancy test as the prototype model for an ideal POCT for STIs. Most focus group members stated they wanted a POCT “*just like a pregnancy test*”, both characteristically and physically.

Participants mentioned that “*ease of use*” was one of the most important characteristics that an ideal POCT should possess. This test should be “*simple to use*” and “*user-friendly*” – i.e. should not require a great amount of technical ability to allow a physician, other clinic staff member or even the patient to run the test without any assistance. The test should be a CLIA-waived test. In addition, the “*test result should be easily read and interpreted with a yes or no when the test is done*”. Accuracy of the POCT was also important for the participants. A POCT with high sensitivity and specificity was desired.

The ideal STI POCT should have a rapid turn-around time. Most participants preferred to have a much shorter turn-around time; as one participant stated “*the quicker, the better*” or another stated “*15 seconds*”. Participants recognized such a short time frame is unlikely, as one participant described “*We wish it could be 5 minutes but that would be impossible.*” The majority's consensus was that 20 minutes is acceptable as the limit of required time but that 5 minutes or less was preferred. One participant stated “*a 30-minute POCT is alright if it can detect chlamydia, gonorrhea, trichomonas, and HIV simultaneously*”.

Participants also preferred the ideal POCT to be compact, durable, and sturdy, the same physical characteristics that the pregnancy test possesses. They specifically stated that the ideal test should not be easily knocked over in a busy clinic setting. Finally, they wished the sample collection to be non-invasive, in order to increase acceptability of the test, especially from the perspective of the patients. Some specific quotations from participants were presented in Table 1.

### Sensitivity, Specificity and Predictive Values

Most participants stated the sensitivity of an ideal STI POCT should be 90% or above, preferably the high 90s, close to 100%, and one participant preferred both sensitivity and specificity as 100%. A few participants would accept a sensitivity of at least 80%. Regarding specificity, the majority wanted it to be similar to the sensitivity, preferably high 90s, close to 100%. Some said the specificity could be traded off a little as compared to the sensitivity. One said that specificity can be low, “*around 75–80%*”. Some participants who did not provide numbers for the sensitivity and specificity stated they would like to have the sensitivity and specificity as high as current tests or as good as nucleic acid amplification tests. Some participants stated that understanding the disease prevalence in the population in which the test would be employed is important, and positive and negative predictive values of the POCT were more important for clinicians than the sensitivity and specificity. A few participants mentioned the specific values of predictive values that they would like to see in an ideal STI POCT. One stated 97% and another stated 80% for a positive predictive value.

## Discussion

To our knowledge, this is the first large-scale in-depth qualitative study illustrating what the clinicians perceive as necessary to make a POCT for STIs practical. Our focus group discussions first documented the barriers encountered or perceived barriers in currently available POCT for STIs, including test-related, clinic operation-related, and patient-related aspects. Discussion session participants further identified the direction for the development of new STI POCTs. They chose chlamydia as the first pathogen for prioritizing the development of a new POCT, and they listed characteristics of an ideal POC diagnostic which would overcome the barriers they identified accordingly. Most of these characteristics have been identified by Mabey and his colleagues 15 years ago [6], indicating there has been little progress on meeting clinicians' needs on the subject of POCT for STIs.

It is not surprising that chlamydia was the preferred choice for a new STI POCT from the 8 structured group discussions. High prevalence and incidence [1], unavailability of a reliable POCT [13], [21], and relative difficulty to diagnose are likely the main reasons that participants overwhelmingly favored chlamydia over other STI pathogens for a new POCT. Some of reasons mentioned above are also likely to explain why herpes simplex virus and *T. vaginalis* were the second and third preferred organisms on the “wish list” for the development of a new POCT. Developing POCTs which can detect multiple pathogens or specific disease condition, e.g. early seroconversion of HIV infection, common syndromes, were mentioned and discussed; however, it appeared they were not the top priority at the current time.

Overwhelmingly, the over-the-counter (OTC) pregnancy test is viewed by all participants as the perfect prototype for an ideal STI POCT, even though the accuracy has been questioned and a faint-colored line has always been an issue for result interpretation [22]. OTC pregnancy test's favorable test performance, operation, and physical characteristics were the same characteristics that the participants would envision an ideal STI POCT to possess. With physical and performance characteristics similar to a pregnancy test, which is well acceptable from both clinicians and patients, a new POCT would be in a good position to be widely acceptable and useful in the clinics.

Complexity of the test, including multiple-step procedures, time-step driven processes, laboratory-driven issues, and difficulty in reading and interpreting the test results, was a major barrier identified by participants during the discussion sessions. Participants indicated that fewer steps, minimal time-measuring steps, a simple dichotomous yes/no or +/− result indicator would make the POCT simple to use and user-friendly in their busy clinic environment. Uniformly, all claimed a CLIA-waived test performed while the patient was in the clinic would be ideal. Thus, this characteristic could reduce the overall turn-around time. In addition, this would allow medical providers to perform testing on patients, or even patients to perform self-testing, initiating a pathway for home testing POCTs that could be used as OTC tests for STIs.

Operation-related barriers, including interruption of clinic work flow and administration regulations, were another main area of perceived barriers. Most of the time, these barriers resulted from the complexity of the test previously described above. All contribute to the interruption of clinic or office work flow. Another important factor was the turn-around time. If turn-around time is longer than the average time allocated for clinician-patient interaction, patients will have to wait, and clinicians will have to interrupt their workup for a new patient in order to give the result to the patient whom they saw earlier. Otherwise, patients have to wait until clinicians have time to disclose the result; which brings up another important barrier from the patient's perspective, and that is a potentially long wait time for the POCT result. Thus, if a new POCT can reduce the complexity of the diagnostic as well as the overall turn-around time, ensuring a feasible and efficient clinic workflow, acceptance of the new test may be probable. Some institutions or clinics may follow a conservative path or may take a longer time when incorporating a new U.S. FDA-approved diagnostic, in part because of concern for potential litigation associated with the use of a new test in their clinical practice. Addressing these issues is beyond the scope of this current project.

Finally, some patient-related barriers affecting patient acceptability to the test were perceived by our clinician participants. These included long wait time, invasiveness of sample collection method, and patients' readiness to receive test results that were not necessarily part of their reason for visit. A new POCT with rapid turn-around time, using non-invasive specimen types such as urine, vaginal swab, or saliva specimens, would minimize most of the barriers in this area. Therefore, to better understand these barriers and the wish list for an ideal POCT, another study has been initiated using the same qualitative approach in patients with or at risk for STIs.

In general, little disagreement was observed during each focus group discussion session. However, some variability was found between sessions, which could result from some specific exposures or experiences that the discussants have had. For example, the rapid syphilis test was only indicated by a professional from United Kingdom and the rapid *T. vaginalis* test was mentioned in two sessions held in Cincinnati. These two sessions also ranked *T. vaginalis as second* preferred organisms for the development of a new POCT.

The main limitation of this study is that our eight focus group discussions might not be representative of opinions of all STI professionals. However, we have tried to include STI experts, directors of 6 U.S. model STD clinics, and clinicians who use and would use new POCTs for STIs from a wide range of clinics where their patients are at high risk for STIs. We feel that, in this exploratory study aiming to discover themes and patterns, and to build initial models of how the ideal POCT should work, our findings provide imperative preliminary evidence of barriers to use of currently available POCTs, priority for development of new POCT, and characteristics of an ideal STI POCT. These results have been used to design a series of survey questionnaires for the industry representatives who are interested in development of new STI POCTs and the clinicians who would use new test.

In conclusion, our study provided substantial information in the formative stage of assessment identifying the need for and perception of qualities imperative for an ideal new STI POCT. Our focus group discussion sessions concluded that chlamydia was considered as the top priority pathogen for development and that ease of use, rapid turnaround and high accuracy were considered as essential characteristics of an ideal POCT. Our findings serve as important guidance for the development of a survey instrument for the end-users and for the direction of the development of a new and ideal STI POCT for use by practitioners working in public health, academia, and industry.
